# Identifying households with children who have complex needs: a segmentation model for integrated care systems

**DOI:** 10.1186/s12913-024-12100-x

**Published:** 2025-01-27

**Authors:** Roberta Piroddi, Andrea Astbury, Wesam Baker, Kostantinos Daras, Joe Rafferty, Iain Buchan, Benjamin Barr

**Affiliations:** 1 Department of Public Health Policy and Systems, University of Liverpool, Brownlow Street, Liverpool, L69 3GF UK; 2 NHS Cheshire and Merseyside Integrated Care Board, 920 Centre Park Square, Warrington, WA1 1QY UK; 3 NHS Mersey Care Foundation Trust, Kings Business Park, Prescot, L34 1PJ UK

**Keywords:** Health service innovation, Population health management, Health needs segmentation, Integrated health and social care systems

## Abstract

**Background:**

Adversity in childhood is increasing in the United Kingdom. Complex health and social problems affecting children cluster in families where adults also have high need, but services are rarely aligned to support the whole family. Household level segmentation can help identify households most needing integrated support. Thus, the aim was to develop a segmentation model to identify those households with children who have high levels of complex cross-sectoral needs, to use as a case-finding tool for health and social care services, and to describe characteristics of identified households, to inform service integration.

**Method:**

Working with stakeholders—in an English region of 2.7m population- we agreed a definition of families having complex needs which would benefit from service integration – including households with high intensity use, who had both physical and mental health problems amongst both adults and children and wider social risks. We then used individual and household linked data across multiple health and social care services to identify these households, providing an algorithm to be used in a case finding interface. Finally, to understand the needs of this segment, and to identify unmet need, to tailor support, we used descriptive statistics and Poisson regression to profile the segment and compare them with the rest of the population.

**Results:**

Twenty one thousand and five hundreds twenty seven households (8% of the population of the region) were identified with complex needs, including 89,631 people (41,382 children), accounting for 34% of health and social care costs for families with children, £362 million in total, of which 42% was on children in care of local authorities. The households had contact with 3–4 different services, had high prevalence of mental health problems, most frequently co-morbid with respiratory problems, with high mental health emergency service use particularly amongst teenage girls many of whom had no prior elective treatment for conditions.

**Conclusion:**

Our model provides a potentially useful tool for identifying households that could benefit from better integration of services and targeted family support that can help break intergenerational transfer of adversity.

**Supplementary Information:**

The online version contains supplementary material available at 10.1186/s12913-024-12100-x.

## Background

In line with global trends, child health is deteriorating in the United Kingdom (UK) [1]. Infant mortality has been increasing at an unprecedented rate over the past decade [2], with the UK is ranked 46th out of the 49 European OECD countries [3]. Mental distress and self-harm are increasing among young people [4]. The number of children placed under the protection of the state has also increased by 20% between 2010 and 2020 [5]. Among general health indicators, British 5-year old boys are on average 7cm shorter than their European counterparts, a remarkable drop of 30 places in national ranks over 10 years [6].

Household health and health-promoting resources are fundamental determinants of child health, and household and parental adversity cluster with adverse outcomes for their children [7]. Adult burden of disease in households [8], and parental mental ill-health influences poor children outcomes [9]. Thus supporting and improving the health of the household is important for protecting and promoting child health [10].

Services are rarely well aligned to support the whole family. Insufficient integration between service providers produces gaps and overlaps in support, and makes it difficult for families to navigate the system effectively [11, 12]. There is an urgent need to provide integrated support to families with complex needs, defined as those presenting multiple clinical, psychological and social needs simultaneously [13, 14]. These families typically require extensive and targeted support due to a specific combination of medical and social challenges, arising from diverse factors such as chronic illness, disability, mental health issues, or difficult life circumstances [15–20].

Risk stratification and segmentation approaches can help identify people at risk to better design services to meet their needs [21, 22]. These approaches have, however, tended to focus on individuals rather than households [23]. Many risks, particularly those related to the wider social environmental (e.g. housing, poverty) act at household level [24, 25]. As well as individuals having multiple needs that cut across organisations barriers (e.g. different disease pathways, health and social care), households may include multiple individuals each with multiple needs [17, 18, 26, 27]. This accumulation means that some households use large numbers of different services, with little coordination between them, high potential for duplication and lost opportunities for synergy.

Approaches to identifying households with complex needs, for targeted interventions, generally focused on narrowly defined high-risk households [28]. For example, the UK Troubled Families programme initially targeted 120,000 families [29] having five out of seven problems (no parent in work; family lives in poor quality or overcrowded housing; no parent with qualifications; mother has mental health problems; at least one parent has a long-standing limiting illness, low income (60% below median); inability to afford food and clothing items) [30, 31]. These approaches have been hampered by a lack of real-time linked data providing information on the breadth of health and social needs [32]. Additionally, they have not applied criteria informed by the need for service integration [33]. However, where needs cut across organisational or service boundaries, this provides opportunities for better integration.

Thus, to provide evidence for integrated services, in consultation with stakeholders, we developed a segmentation model for households with complex needs across Cheshire and Merseyside (C&M), a region in the North West of England. Using newly available routine linked data, this model aims to identify households that could benefit from integrated support, creating an indicator for local practitioners’ case finding tools. Additionally, this work aims to profile these households’ characteristics and service use to inform the design of new integrated services in high-demand sectors, to improve outcomes for all household members and particularly children.

## Methods

### Developing the complex households definition

We developed the definition iteratively through a series of workshops with stakeholders and data analysts. We aimed for a simple model for identifying households with complex needs using newly available routine linked data, providing a live view of patterns of service use of these households for local practitioners to use to target support. We focused on households who experience multiple health and possibly social challenges and use multiple different services, as a basis for actions to better integrate these services around households needs.

Stakeholders in these workshops were: health and social care planners at region and district levels, public health practitioners, primary care doctors, health and social care providers organisations (the English national health service or NHS, municipalities and charity and voluntary sector), and people with lived experience of complex lives, alongside analysts and academics experiences in the use of electronic linked data. In total there were 6 workshops between September 2021 and March 2023.

**Fig. 1:**
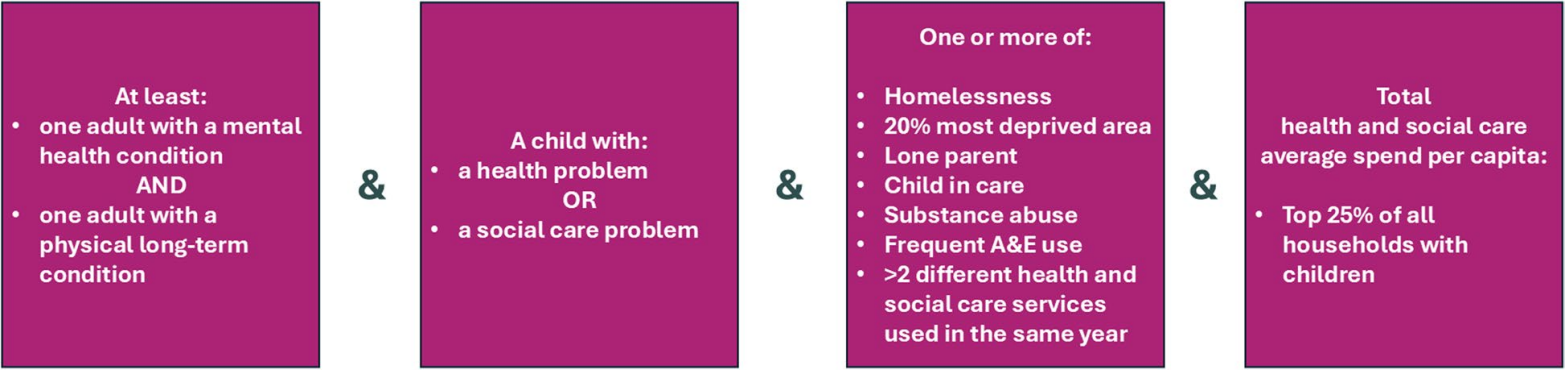
Definition of households with children who have complex needs

The first workshop focused on the review of datasets available for analysis and discussed an initial analysis of population health needs obtained using an existing segmentation model of individual complex health needs [23]. Participants shared views on defining household complexity using linked datasets, and prioritised focusing on households with children, health issues, and health-social care interactions. A technical workshop clarified measurable data. Further workshops discussed service integration elements, initial definitions, and exploratory findings. Public engagement workshops then interpreted findings and highlighted priorities of people with lived experiences. This process is illustrated in Appendix A. These iterations converged to a definition: households with children (aged 0–16) that include adults that have a combination of mental and physical health problems and at least one child with a known health or social care problem, an indicator of potential social problems, and average per capita household cost-weighted health and social care utilisation in the upper quartile of all households with children (as in Fig. 1).

### Data sources

We used linked individual level data for all 2.7 million people registered with family doctors in a region of North West of England, Cheshire and Merseyside (C&M), in 2021. We used electronic administrative records from primary care [34], secondary care [35, 36], mental health services [37], community services [38], social care [39], mortality records from the Office for National Statistics (ONS) [40], and small-area aggregated measures of multiple deprivation called Index of Multiple Deprivation (IMD) [41]. Details of each dataset are given in Appendix B. We excluded 3% of people whose doctor had no data sharing agreement. These datasets were pseudonymised and linked by the NHS Data Service for Commissioners Regional Offices (DSRCO), before being made available for analysis through a secure data environment.

### Variables

We defined a set of variables related to individual health and social care needs including diagnostic flags and service activity in 2021 (Appendix C provides a complete list of variables included and Appendix D clinical codes and corresponding definitions). A pseudonymised household indicator was also made available with the data. This was derived by linking patients’ addresses in mid-2021 to Unique Property Reference Numbers (UPRN) [42]. This UPRN was then pseudonymised providing an indicator in the data showing which records refer to people living at the same address. To define households, we then assumed that people with the same anonymised UPRN constituted the same household. We excluded people who were living at the same address with more than 10 people, as these potentially reflected people living in institutions (e.g. care homes), houses of multiple occupation and other addresses that might include multiple households. The threshold of 10 reflects levels of supervision and characteristics of communal living facilities in the UK [43].

### Deriving the complex households segment

To derive the complex household segment, we used four conditions that must be satisfied at the same time, as in Fig. 1.

#### Condition 1

Adults with both physical and mental long-term conditions. Physical conditions include asthma, cancer, cardiovascular disease, chronic kidney and liver diseases, chronic obstructive pulmonary disease, dementia, diabetes, epilepsy, gastroenterological, neurological, rheumatological diseases, and physical disability. Mental conditions include anxiety, depression, severe mental illness (e.g., schizophrenia, psychosis), neurodevelopmental conditions (e.g., autism, attention deficit and hyperactivity disorder (ADHD)), and learning disabilities.

#### Condition 2

A child with any health or social problems, including all long-term conditions and disabilities listed for adults, or an indication that the child is under state protection (child in care).

#### Condition 3

Indicators of social vulnerability, such as homelessness, living in the most deprived 20% of the municipality, lone parent households (where only one adult resides), a child in care (i.e. children where the state is the legal guardian and they do not live with their original family), substance abuse, frequent Emergency Room (accident and emergency or A&E in UK) visits (more than 5 per year), and use of 3 or more different health or social care services.

#### Condition 4

Households in the top 25% of per-capita health and social care expenditure for the area. We derived costs: in primary care as an estimate of the spend for the management of different chronic conditions [44], in secondary care from the national tariff applied for each contact included in the routine data [45], for mental and community mental health services from the reference national costs [46], for adult social care from the published regional spend statistics [47], for children social care from averaged published total area spend [48].

Detailed methods for the data extraction and processing, including cost calculations, are in Appendix C. All code and algorithm descriptions are available in our GitHub public repository [49].

### Analysis

Applying the four conditions above with the algorithm described in Appendix C, we created an indicator to identify households for targeted support. To understand the needs of the segment and which services exhibited the best potential for integration, we used comprehensive descriptive statistical analysis to profile the clinical and utilization characteristics of the complex household segment, juxtaposed with all other households with children in the region. Descriptive statistics were employed to delineate density distributions of age and sex, alongside histograms of composition of households by age group. We examined the prevalence of conditions stratified by age group and sex. We analysed the frequency of incident events in different health and social care sectors. To explore the potential for service integration, we assessed the presence of comorbidities and the distribution of service utilization co-occurring across different service boundaries. Furthermore, we used Poisson regression to compare level risks between complex households and other households. For example, to investigate potential unmet need we compared the risk of emergency use of services with and without previous engagement with community routine services. This was proxy measure motivated by the inverse care law [50], as people who have larger need tend to have less access to services and then experience more crises requiring emergency service use [51].

## Results

Two million, six hundreds forty five thousand and three hundreds twenty nine individuals were registered in 2021 with a doctor in C&M were included in the analysis. 1,022,840 individuals lived in a household with at least one child aged 0–16. Based on our definition, 21,527 households with children (8% of all households with children) were identified as having complex needs including 89,631 people (of which 41,382 children). This 8% of families accounted for an estimated 34% of health and social care costs for families with children in C&M, £362 million in total.

Table 1 shows the proportion of households with children in C&M that satisfy each of the four criteria in Fig. 1. Other households with children, on average, have fewer adults with physical-mental co-morbidity, fewer children with health or social problems, and use fewer NHS and social care resources. The table shows the main differences in the vulnerabilities included in criteria 3 for the complex households are: higher use of urgent care, higher levels of drug and alcohol problems, more children in care. Almost all the complex households use more than two different services.

**Table 1:** Proportion of households with children in C&M that satisfy each of the four single criteria that need to be satisfied at the same time for a household to be included in the segment of complex households

Criteria	Total number of households with children in C&M		Other households		Complex households	
	*N* = 266,939		*N* = 245,412		*N* = 21,527	
	Number	%	Number	%	Number	%
1- At least one adult with physical long-term condition AND at least one adult with a mental health condition	74,731	27	53,204	22	21,527	100
2- A child with a health problem or a social care problem	96,853	36	75,326	30	21,527	100
3- One of more of the socio-economic vulnerabilities listed below:	161,264	60	139,737	57	21,527	100
• Homelessness	92	0.03	71	0.03	21	0.1
• High A&E (> 5 per year)	2189	0.8	1472	0.6	717	3
• Substance abuse	16,089	6	11,714	5	4375	20
• Lone parent	59,106	22	53,661	22	5445	25
• Child in care	4528	1.7	4528	1	2043	9
• Deprived	53,373	20 (by definition)	47,001	19	6372	30
• More than 2 different services	96,713	36	76,912	31	19,801	92
4- The average total per capita spend is in top quarter of total health and social care spend in the region for households with children	66,698	25 (by definition)	45,171	18	21,527	100

### Characteristics of the segment

Table 2 shows the demographic characteristics and prevalence of long-term physical and mental health conditions in children and adults in complex needs households compared to the other households. Members of households with complex needs were more likely to be of white ethnicity (93%) than households without complex needs (89%). Households with complex needs have high levels of long-term health conditions, with 50% of people have at least 1 long term condition, 20% having 2 or more and 6% having three or more. The most common comorbidity is asthma and depression/anxiety, affecting more than 4% of this population compared to 0.7% in other households.

**Table 2:** Characteristics of children and adults living in complex households compared to other households: demographics and prevalence of disease

Characteristics:		
Children (0–16 years old) in:	Other households	Complex households
N	413,661	41,382
Age (mean (SD))	8.15 (4.77)	9.54 (4.65)
Sex = M (%)	51.3	51.7
IMD (mean (SD))	0.2 (0.16)	0.25 (0.16)
Asthma (%)	6	20
Epilepsy (%)	1	4
Neurodevelopmental and learning disabilities (%)	2	12
Anxiety and/or depression (%)	3	13
Adults in:	Other households	Complex households
N	517,284	48,249
Age (mean (SD))	39.18	39.48
Sex = M (%)	44.9	42.3
Asthma (%)	15	26
Chronic obstructive pulmonary disease (%)	0.1	2
Cardiovascular disease (%)	2	4
Cancer (%)	1	2
Diabetes (%)	3	7
Epilepsy (%)	3	6
Chronic kidney disease (%)	1	2
Chronic liver disease (%)	2	4
Neurodevelopmental and learning disabilities (%)	1	4
Anxiety and/or depression (%)	6	20
Severe mental illness (%)	1	3

**Fig. 2:**
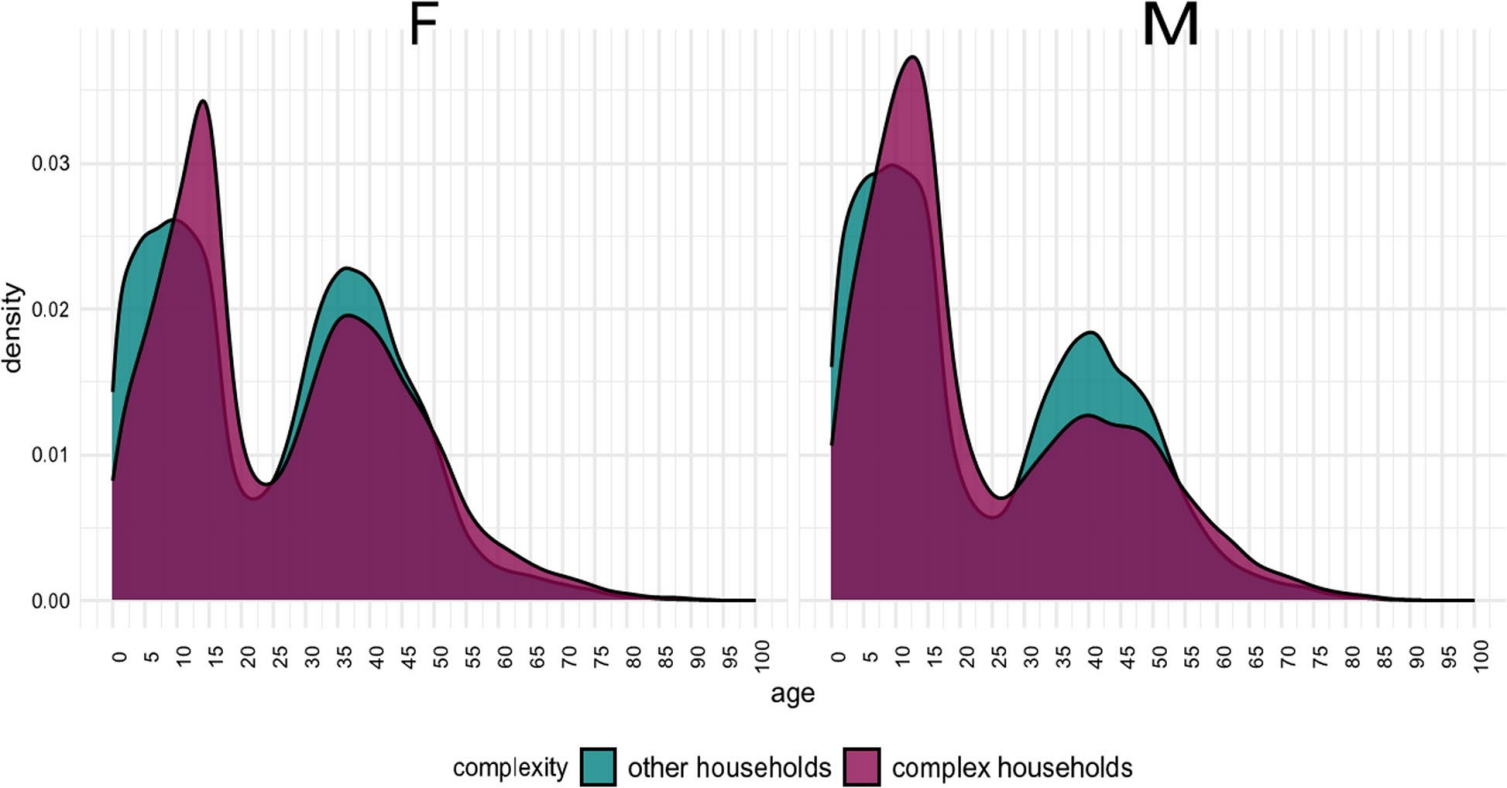
Age/sex distribution of members of households with complex needs and members of other households with children

Figure 2 shows the age and sex distribution. Households with complex needs, tended to have more teenage children and fewer adult men than other households. Households with complex needs also tended to be larger than other households (mean household size = 4.2, versus 3.7).

### Resource utilization and spend distribution

**Fig. 3:**
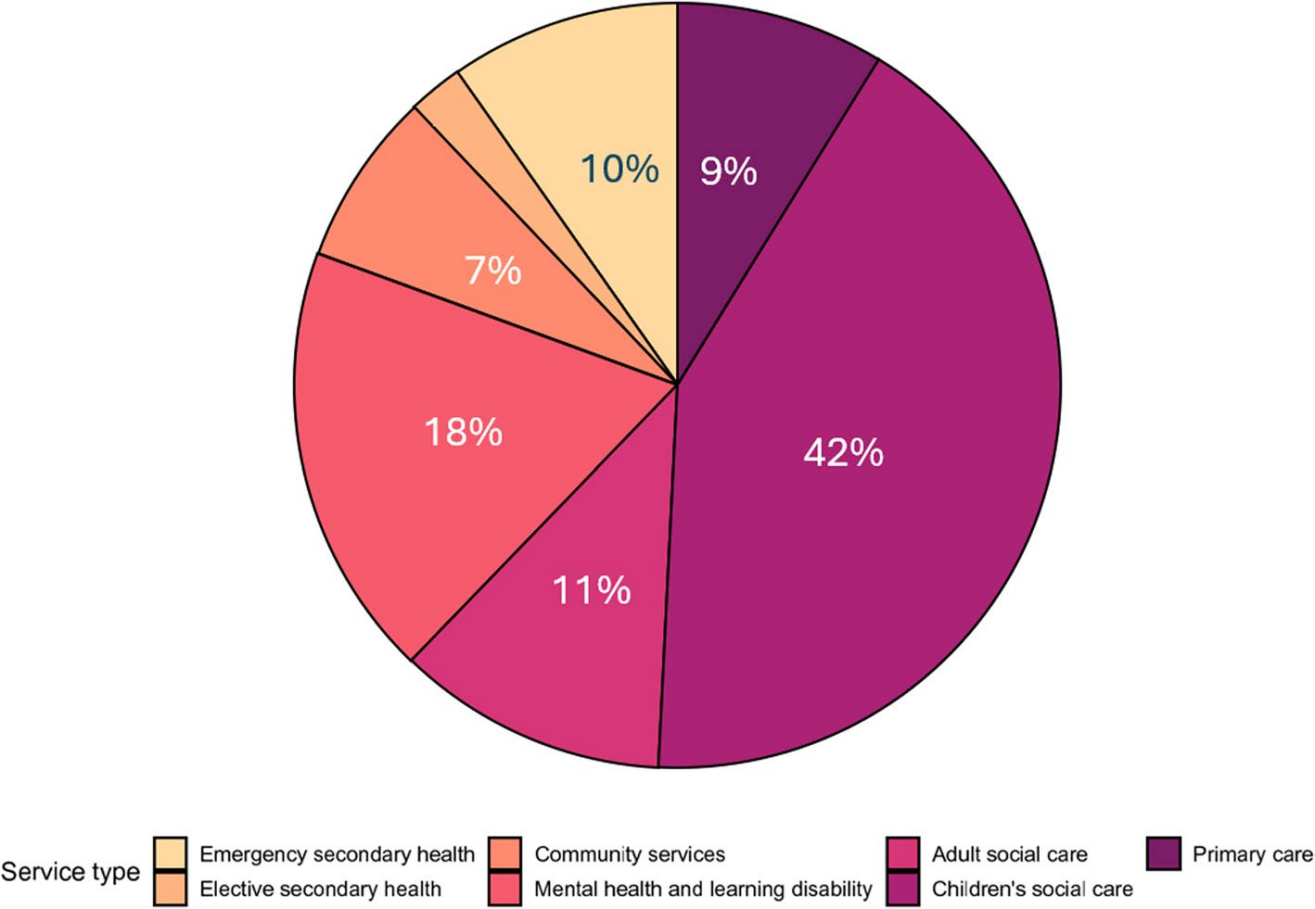
Distribution of total health and social care expenditure by service type for members of households with children who have complex needs in C&M

Figure 3 shows the estimated distribution of costs by service type for households with complex needs.

Applied to the region of C&M and for the cost of health and social services used only for households with children aged 0–16, 42% of the total estimated health and social care spend for these households was due to funding for children placed under the protection of the state (children’s social care). Contacts with mental health services accounts for 18% of the total spend on health and social care services by households with children who have complex needs.

Complex households use multiple services with 90% of households using three or more types of health and social care service.

Appendix E describes the intersection of service types used. As the aim of the work was to identify which services to integrate, it is useful to understand which combinations of services are used more frequently by households with complex needs. These households are characterised by high co-use of accident and emergency, community services (including health visiting) and mental health services. Of the non-emergency services – there was high co-use of services between primary care, community services and mental health services.

### Emergent vulnerabilities and unmet need

55% of households with complex needs had used or been referred to community mental health and learning difficulty services in 2021 (compared to 6% for other households).

**Fig. 4:**
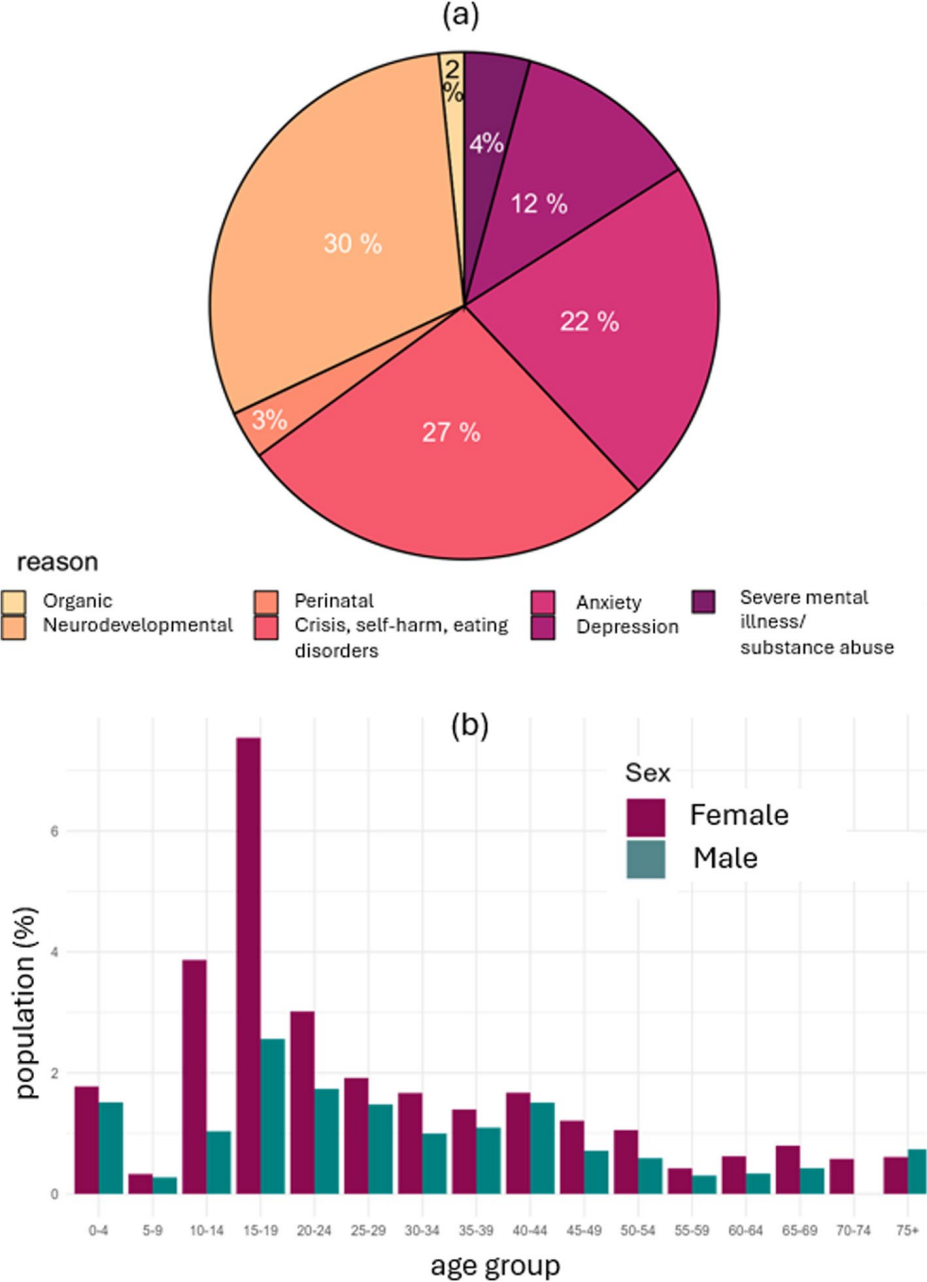
Use of community mental health services subdivided by reason for contact (**a**) and emergency room (A&E) yearly attendance for mental health reasons for the people in households with complex needs by age group and sex (**b**)

Figure 4a shows different types of mental health services used by households with complex needs. They were particularly likely to use services for anxiety, crisis, self-harm, eating disorders, neurodevelopmental disorders and autism. Emergency Room (A&E) attendances for mental health problems were particularly high for teenage girls from households with complex needs, as in Fig. 4b.

To understand potential unmet needs, we investigated emergency attendances at hospital (A&E or hospital admission) for mental health reasons from people who had no previous contact with mental health services or mental health diagnoses in primary care. Emergency attendances in this group were 7 times higher (OR = 6.8, 95%CI 6.5 to 7.1) for people living in households with complex needs compared to those who were not. In contrast for people already known to services with mental health problems, emergency attendances were only 2 times higher in complex need households compared to non-complex need households (OR 2.0, 1.9 to 2.1). This suggests potentially high levels of unmet mental health needs in these households, leading to high emergency use.

## Discussion

Our model identifies 8% of households with children in 2.6 million population as having complex needs. This was a much larger percentage compared to other models currently used for targeted support [29]. These households were larger and had more adolescent members that other households. The greatest expenditure was on children’s social care (42%). From the point of view of opportunities for integration, a notable characteristic of these households was the high prevalence of comorbid of mental and physical health (especially respiratory) conditions in both adults and children. Carers of children experiencing complex physical disabilities are at high risk of poor mental health and struggle with maintaining functional family life [19, 52]. Children who experience chronic health conditions also are more at risk of anxiety and depression [53, 54], including into adulthood [55, 56]. Poor parental mental health affects children mental health [26, 57, 58], and research on perinatal and maternal mental health found it affects child physical health [27]. More research is needed to explore the complex interaction of physical and mental health in carers, not just mothers. Respiratory ill-health, specifically asthma, which is strongly related to socioeconomic position [59, 60], and is the most common long term physical condition in children [61], may be a priority area. In adults, there was a high prevalence of severe mental illness, and in children a high prevalence of learning disabilities, difficulties, and neurodevelopmental conditions. Households with complex needs used more types of services than other households and had higher uses of emergency secondary care and community mental health services (30% of these services were for crises and self-harm). Use of emergency services for a mental health reason was very high in children and young adults, particularly adolescent girls and young women. The high burden of adolescent mental health distress is important as poor parental mental health is a strong risk factor for negative physical and mental health outcomes in children and increases risk of being taken into care [7, 9, 26, 27, 57, 62]. We also know there is a crisis in adolescent mental health [63–66] and a marked exacerbation of mental distress especially in adolescent girls [67–69]. Overall this indicates the presence of unmet need and the opportunity for proactive intervention in adolescents in this segment.

### Strengths and limitations

Existing population health segmentation methods fall into two categories [70]: one of rule-based tools using expert clinical judgment [71]; and data-driven tools using real-world data [72]. Our approach combines both. Integrating cross-sectoral data provides system-wide insights. Leveraging large-scale health and social care data reveals some population health interactions, enabling tailored interventions for households. Stakeholder engagement ensures alignment with local priorities [73]. Finally, the focus on households rather than individual patients represents a significant departure from traditional individual-centric approaches [74]. By considering the collective health needs of households, this model acknowledges the interconnectedness of family members and their shared health environment.

There are multiple limitations and potential improvements for this model. Firstly, we used data of healthcare contacts to define complexity. Because of the inverse care law, those who most need healthcare also face the biggest barriers to access services [50]. Relying on service and diagnostic data risks excluding those with high complexity, like the homeless, refugees, asylum seekers, or travellers. Our data likely misses or has incomplete information on these groups. Cross-sectoral data helps mitigate this limitation somewhat. Populations with poor healthcare access often use emergency care extensively, which we used to identify unmet needs. Triangulating cross-sectoral data also helped us identify children in state care, one of the most vulnerable groups. We acknowledge that to reduce inequalities risk in this tool, we needed to have more data on social determinants such as welfare, housing, education, employment, crime, and environment.

Secondly, we excluded data from 3% of the population whose doctors did not share data. These individuals were concentrated in a more affluent, older area, likely contributing fewer households with children and even fewer with complex needs. Thirdly, using the UPRN to identify households, based on a 2021 snapshot may miss people who have moved since then. Sharing an address does not imply sharing a household, and this research may not identify multiple households at a single-family address. UPRNs are typically linked to one household unless it is a multi-occupancy building, so multiple households at a single-occupancy address would be unusual [75, 76]. However, practitioners using the tool would need to check whether people at the same address are actual single families. Fourthly, we estimated costs using rough calculations. We had accurate costs on secondary care, and we estimate that primary and mental health costs might be an under-estimate. Therefore complex households may be accounting for a larger percentage of the total expenditure than in this analysis, as they use these services more than the rest [77]. Finally, we identified children in state care from health records, rather children’s social care, where we could not access individual level data. The numbers of children in care matched the aggregate numbers published by municipalities [48]. However, we missed data about other children known to social care but not in care of the state, for example those who have special care or protection plans.

### Policy implications

This approach may be useful for resource allocation to communities with high concentrations of households with complex needs, especially if in the future datasets contain information on social and environmental determinants of household health. It supports the development of targeted mental health services, including parental support during adolescence. Co-locating services with primary care could enhance prevention efforts. Clusters of respiratory and mental comorbidity suggest integrating mental health care within physical health teams. This kind of integration – embedded within primary care—has shown improved mental as well as physical health outcomes, with notable implementations in low as well as high resource settings [78, 79].

Traditional policies often operate in silos, separating mental health and physical health services. But the identification of a substantial comorbidity burden between mental and physical health within households underscores the need for integrated care approaches. Policymakers should prioritize initiatives that bridge the gap between mental health services and general healthcare. Fostering collaboration between primary care providers, mental health specialists, and social services, integrated care services can enhance early detection, improve outcomes, and reduce overall healthcare costs.

Being taken into care of often has adverse outcomes for the children and places a major financial burden on public services. For example, UK municipalities are struggling financially, with costs of children placed in their care a major contributor [80]. Our model identified families with likely high needs for children’s social care interventions, which may reduce escalating risks.

Policymakers should encourage data sharing across health and social care sectors. Interoperability and linkage between electronic health records, social services databases, and educational institutions could enhance the accuracy of household-level segmentation. Collaborative efforts would allow for evidence-based policy development and targeted resource allocation.

Our model identified 8% of households in a large English integrated care system as having complex needs. Existing funding models often prioritize high-risk individuals and a very small percentage of the population experiencing extremely complex and seemingly intractable social problems and severe crises [31, 81]. But this segmentation and the household focus encourage a shift towards a wider and more preventive approach, recognising that comorbidity affects entire households, but also that it is possible to identify clusters of needs that may be addressed simultaneously and for several members of the same household, multiplying the benefit.

In terms of sustainability, there remains significant scope for savings by reducing inter-organisational duplication. In this study, we have demonstrated a mechanism to achieve efficiency from scarce resources by allocating the right mixture of healthcare to maximise the welfare and health outcomes of communities. It is an alternative to technical efficiencies created, for example, by cost-saving decisions [82].

## Conclusions

Our segmentation identifies families with complex service use patterns, aiming to improve outcomes through better service integration. This model was implemented in an English regional health system as an indicator for case-finding tools for practitioners, helping providers target support and redesign services. Households with complex needs often have high levels of mental and physical health issues, especially respiratory problems, and use both children’s social care and mental health services extensively. Teenage children in these households have particularly high mental health needs, often poorly managed, leading to high emergency care use. Addressing mental health problems and learning difficulties in these teenagers can potentially break the cycle of intergenerational adversity.

## Supplementary Information

Supplementary Material 1.

Supplementary Material 2.

Supplementary Material 3.

Supplementary Material 4.

Supplementary Material 5.

## Data Availability

Data are currently available under licence from NHS England. Statistical code and business rules are available in our GitHub public repository. (https://github.com/cipha-uk/complex_households).

## Abbreviations

A&E: Accident and Emergency, Emergency Room/Department denomination in the UK
C&M: Cheshire and Merseyside, a region of the UK, in the North West of England
DAAG: Data Access and Approval Group
DSCRO: Data Services for Commissioners Regional Office
IMD: Index of Multiple Deprivation
NHS: National Health Service, UK’s state funded universal health service
ONS: Office for National Statistics
OR: Odds ratio
UPRN: Unique Property Reference Number
