# Supplementary material for: Identifying households with children who have complex needs: a segmentation model for integrated care systems
Source: BMC Health Serv Res. 2025 Jan 27;25:152. doi: 10.1186/s12913-024-12100-x (PMC11773761; doi:10.1186/s12913-024-12100-x)
Supplement: Supplementary file 1 — Supplementary Material 1. [file 12913_2024_12100_MOESM1_ESM.docx]

**Identifying households with children who have complex needs: a segmentation model for integrated care systems**

**Supplemental materials: Developing the definition with an iterative and collaborative approach**

**Ways of working:** Cheshire and Merseyside health and care system partners work collaboratively to use data to drive innovation and improvement in population health and care using networked analytics teams that were piloted through embedded researcher projects, e.g. the Networked Data Labs. Networked analytics teams are groups of data analysts and scientists from different departments in an organisation and different organisations, working together on the same data sources to harness their collective knowledge and expertise for problem-solving and decision-making. In the case of Cheshire and Merseyside, the networked analytics team comprises of academic researchers from multiple departments, and intelligence analysts from different health and social care organisations, such as the organisations that plan and pay for services (NHS commissioning groups for health and local government for social care) and the organisations who provide services (e.g. acute trusts). Because of the sensitivity of the data, the teams use robust data infrastructure to ensure data is handled in accordance to privacy laws and safeguarding measures are in place, while at the same time allowing analysts to collaborate. In Cheshire and Merseyside such infrastructure is the NHS Secure Data Environment (SDE.) The SDE is a highly secure platform designed for storing and accessing health and social care data. It allows approved users to access and analyze data without the data ever leaving the secure environment. Data access is controlled to approved users. Data is pseudonymised to protect privacy. SDEs are part of a network of infrastructure for data sharing across platforms and organizations.(4,5)

**Stakeholders:** Cheshire & Merseyside is a region which covers 9 municipalities. Municipalities in UK provide public services, e.g. housing, planning refuse collection, for a given geographical area. They are also responsible for commissioning social care services for adults and children and for public health. Stakeholders in this project were: the commissioners (i.e. the planners and funders) of health care services, i.e. the NHS , and the commissioners of social care and public health services, specifically the public health departments of these municipalities. Other stakeholders included the providers of services: there were several acute trusts – i.e. hospitals – including a very large hospital specialising in child health, the two trusts providing all mental health services for the region, one large provider of community health services. With the progression of the project, several voluntary and charitable organisations also were involved. Within the stakeholder organisations, networks of specialists in mental health, children’s health, special educational needs and learning disabilities participated to co-production events. In total, this involved 45 different organisations.

Other categories of stakeholders included analysts and academics to provide insights from data and people of lived experiences of complex needs in their households and organisations and networks of public advisors to provide interpretation of analysis in co-production workshops.

Figure 1: Timeline of key workshops and interactions.


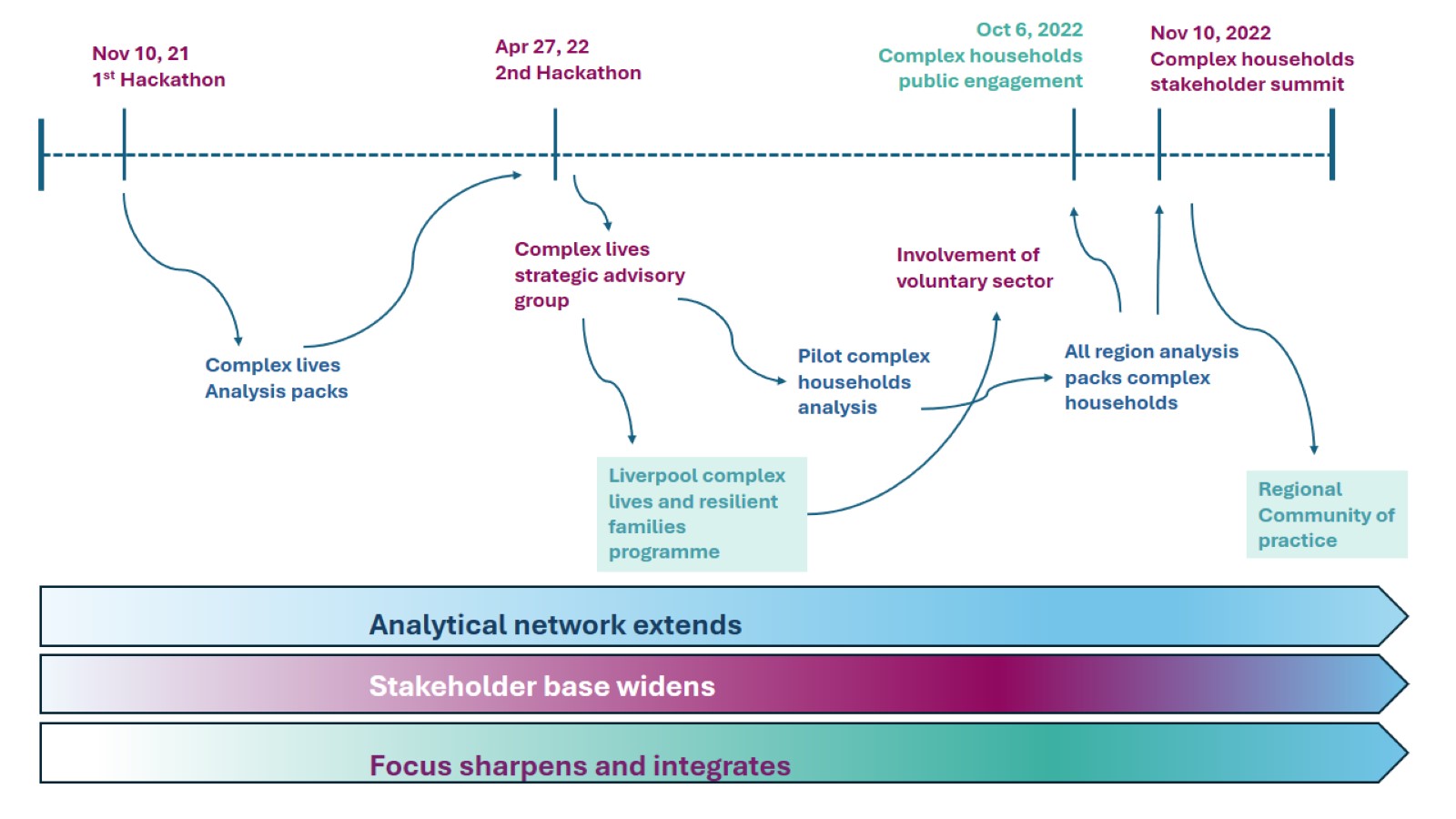


**Workshops:** Workshops tended to have different emphases and mixture of participants. There would be workshops more focussed on analytical views and others dedicated to gathering insight from lived experts. Stakeholders would commission exploratory analysis of the analytical teams and then convene a corresponding public engagement workshop where analytical findings would be interpreted and queried. Stakeholders would then synthetise conclusions from both sets of workshops and refine requests for further iterations. Figure 1 contains a diagrammatical representation and timeline of salient interactions. During the project the analytical team network and the stakeholder base expanded. The focus refined also with the input from the public involvement. In particular, the original stakeholders’ focus was on individuals who present complex needs, to consider families or households with children, based on the feedback from the public that the individual definition of complex needs was too narrow, that origins of long-lasting difficulties may be traced in adversity experience in families and that properly supported families may be an effective vehicle for prevention of escalations. Following the change in focus, stakeholders for the charity and voluntary sectors were also involved, as the issue of trust between public and institutions emerged as a theme, and a community of practice established to further the activity through a reflecting on system-change.

Through this iterative process we aimed to achieve coherence and cognitive participation in our collaborative network, as pre-requisites to embed system change.(6)

The interactions with people with lived experiences showed that issues of imbalance of power and trust in institutions needed more time. After the end of the workshops in March 2023, the commissioners of this project obtained additional funding for further patient and public involvement and engagement activities to continue this work.

References:

1. Improving children and young people’s mental health services - The Health Foundation [Internet]. [cited 2024 Sep 23]. Available from: https://www.health.org.uk/publications/reports/improving-children-and-young-peoples-mental-health-services

2. Assessing the impact of COVID-19 on the clinically extremely vulnerable population - The Health Foundation [Internet]. [cited 2024 Sep 23]. Available from: https://www.health.org.uk/publications/reports/assessing-the-impact-of-covid-19-on-the-clinically-extremely-vulnerable-population

3. Piroddi R, Downing J, Duckworth H, Barr B. The impact of an integrated care intervention on mortality and unplanned hospital admissions in a disadvantaged community in England: A difference-in-differences study. Health Policy. 2022 Jun 1;126(6):549–57.

4. GOV.UK [Internet]. [cited 2024 Sep 24]. Secure data environment for NHS health and social care data - policy guidelines. Available from: https://www.gov.uk/government/publications/secure-data-environment-policy-guidelines/secure-data-environment-for-nhs-health-and-social-care-data-policy-guidelines

5. NHS England Digital [Internet]. [cited 2024 Sep 24]. Secure Data Environment. Available from: https://digital.nhs.uk/services/secure-data-environment-service

6. May C, Finch T. Implementing, Embedding, and Integrating Practices: An Outline of Normalization Process Theory. Sociology. 2009 Jun 1;43(3):535–54.
