# Supplementary material for: Identifying households with children who have complex needs: a segmentation model for integrated care systems
Source: BMC Health Serv Res. 2025 Jan 27;25:152. doi: 10.1186/s12913-024-12100-x (PMC11773761; doi:10.1186/s12913-024-12100-x)
Supplement: Supplementary file 2 — Supplementary Material 2. [file 12913_2024_12100_MOESM2_ESM.docx]

**Identifying households with children who have complex needs: a segmentation model for integrated care systems**

**Supplemental materials: Data sources and description**

This project was set in the English region of Cheshire and Merseyside, in the Northwest of England, in UK. Cheshire and Merseyside has a population of 2.5 million residents.

We used linked data from electronic health records collected for administrative purposes in the English National Health Service (NHS) settings of primary care,(1) secondary care,(2,3) mental health services,(4) community services,(5), with: electronic administrative social care records from municipalities (there are 9 municipalities in the English region where this project took place, covering the local government administrative functions for all the territory),(6) mortality records from the Office for National Statistics (ONS),(7) and small-area aggregated measures of multiple deprivation called Index of Multiple Deprivation (IMD),(8) for the population of registered with a NHS general practice (GP) in 2021.

Appendix C gives the list of variables we used and their description. In general, the primary care datasets contain data of all patient interactions in with their family doctor in primary care: we used these records to looks for diagnoses of groups of long-term conditions, (e.g. cardio-vascular disease, asthma, depression practice) and for prescriptions of certain drugs (e.g. antidepressants).(1) Secondary care datasets contain records of all patient interactions with acute hospitals: we used these records to count emergency and elective admissions(3) and the attendance to emergency departments(2) for all causes and for specific mental health reasons and their cost. Mental health care datasets (4) record the interactions of patients with mental health services in the community and inpatient mental health facilities: we used these datasets to count the referrals to clinics and their reason for referrals and the contacts with services and their type (e.g. eating disorders, autism, first episode psychosis, etc.). Community health datasets (5) contain the information of the interaction of patients with community health services: these are services that are provided in a patient’s home or in community clinics, schools or care homes. We used these datasets to count the number of referrals and contacts to community health services and which clinics they used (e.g. health visitors for parent of young children, physiotherapy). Adult social care services in the UK are services to help people with their day-to-day life in their old age or if they have a disability or recovering from illness. These services are available from public funds only to the people who have the highest needs and the lowest assets. Local government assesses the eligibility and releases funds, whereas the services are provided by a mixture of providers. The adult social care data we used contain the interactions of people who received support funded by public money only.(6) Typical services may help with personal care at home, rehabilitation, residential and nursing home care. We used this data to count the number of referrals and contacts with these services and the type of service received. The English Index of Multiple Deprivation (IMD) is a publicly available statistic widely used in the UK policy and administration: it measures relative deprivation. It contains seven domains of deprivation (income, employment, health, education and skills, housing and services, crime, environment) that are weighted and averaged over small administrative neighbourhoods (1000 people on average) to make the whole measure. In England it is published by the Department for Housing, Communities and Local Government and all the four nations in the UK are responsible for publishing their own index.(8) We used the 2019 version of the IMD, as the values are periodically re-calculated.

References

1. NHS England » Purpose of the GP electronic health record [Internet]. [cited 2024 Oct 28]. Available from: https://www.england.nhs.uk/long-read/purpose-of-the-gp-electronic-health-record/

2. NHS England Digital [Internet]. [cited 2024 Oct 28]. Emergency Care Data Set (ECDS). Available from: https://digital.nhs.uk/data-and-information/data-collections-and-data-sets/data-sets/emergency-care-data-set-ecds

3. NHS England Digital [Internet]. [cited 2024 Oct 28]. Secondary Uses Service (SUS). Available from: https://digital.nhs.uk/services/secondary-uses-service-sus

4. NHS England Digital [Internet]. [cited 2024 Oct 28]. Mental Health Services Data Set (MHSDS). Available from: https://digital.nhs.uk/data-and-information/data-collections-and-data-sets/data-sets/mental-health-services-data-set

5. NHS England Digital [Internet]. [cited 2024 Oct 28]. Community Services Data Set (CSDS). Available from: https://digital.nhs.uk/data-and-information/data-collections-and-data-sets/data-sets/community-services-data-set

6. NHS Arden&GEM. Adult Social Care Client Level Data. [cited 2024 Jun 7]. Adult Social Care Client Level Data. Available from: https://www.ardengemcsu.nhs.uk/adult-social-care-client-level-data/

7. Office for National Statistics. ons.gov.uk. [cited 2024 Jun 7]. Mortality statistics in England and Wales QMI - Office for National Statistics. Available from: https://www.ons.gov.uk/peoplepopulationandcommunity/birthsdeathsandmarriages/deaths/methodologies/mortalitystatisticsinenglandandwalesqmi

8. Department for Levelling Up, Housing and Communities. GOV.UK. [cited 2024 Jun 7]. English indices of deprivation 2019. Available from: https://www.gov.uk/government/statistics/english-indices-of-deprivation-2019
