# Supplementary material for: Identifying households with children who have complex needs: a segmentation model for integrated care systems
Source: BMC Health Serv Res. 2025 Jan 27;25:152. doi: 10.1186/s12913-024-12100-x (PMC11773761; doi:10.1186/s12913-024-12100-x)
Supplement: Supplementary file 3 — Supplementary Material 3. [file 12913_2024_12100_MOESM3_ESM.docx]

**Identifying households with children who have complex needs: a segmentation model for integrated care systems**

**Supplemental materials: algorithm and rules**

1. **Algorithm:**

The algorithm to identify the households with complex needs is described in the flow diagram in Figure 1.

Figure 1: Algorithm flow diagram.


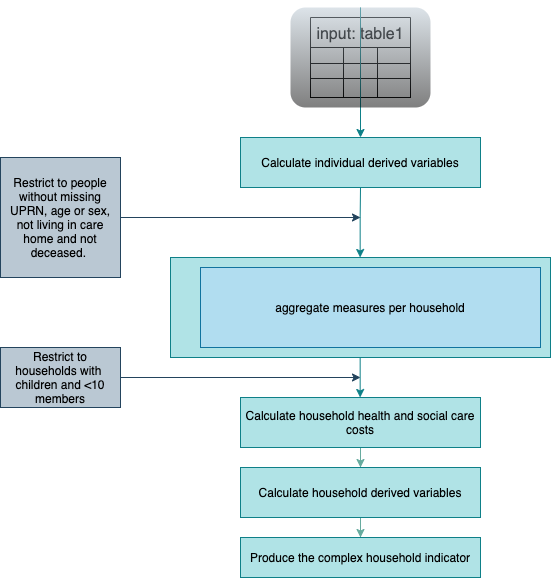


1. **Input variables:**

Table 1 lists the variables in input to the segmentation algorithm to identify households with complex needs.

Table 1: Variables extracted from cross-settings datasets and linked by common pseudonym: these were the inputs to the algorithm to segment the population.

| **variable** | **description** | |
| --- | --- | --- |
| pseudonymised_NHS_Number | Linking pseudonym common in all data sources | |
| pseudonymised_UPRN | UPRN, indicating addressable building of residence– recorded in middle of period considered | |
| characteristics variables |  | |
| from primary care records |  | |
| gp_practice_code | Primary care practice identification number | |
| place_of_registration | Place of primary care registration | |
| place_of_residence | Place of residence | |
| age | Age in years in the middle of period considered | |
| sex | Sex (from birth or registration) | |
| deceased | A flag indicating if the person is dead at beginning of period considered | |
| lsoa | Small area reference code (from residential address – withheld from data) | |
| income_child_score | Income deprivation affecting children (IDACI) from the English index of multiple deprivation 2019 (from lsoa) | |
| nursing_carehome_flag | resident of nursing or residential care home flag (0/1) | |
| homeless | homelessness status flag (0/1) | |
| carer | Informal carer status flag (0/1) | |
| child_looked_after | child is or has been looked after flag (0/1) | |
| substance_misuse_gp | Flag indicating psychoactive substance misuse in primary care records (0/1) | |
| cancer | Flag indicating any cancers 2 years diagnosed in primary care record | |
| learning_disability_pc | Flag indicating any learning disability in primary care record | |
| physical_disability_pc | Flag indicating any physical disability in primary care record | |
| CMHP | Flag indicating diagnosis with any common mental health problem in primary care record. | |
| SMI | Flag indicating diagnosis with Severe mental illness in primary care record. | |
| CVD | Flag indicating diagnosis with CVD in primary care record. | |
| diabetes | Flag indicating diagnosis with Diabetes in primary care record. | |
| gastroenterological | Flag indicating diagnosis with Gastroenterological condition in primary care record. | |
| rheumatological | Flag indicating diagnosis with Rheumatological in primary care record. | |
| epilepsy | Flag indicating diagnosis with Epilepsy in primary care record. | |
| CKD | Flag indicating diagnosis with CKD in primary care record. | |
| CLD | Flag indicating diagnosis with CLD in primary care record. | |
| asthma | Flag indicating diagnosis with Asthma in primary care record. | |
| COPD | Flag indicating diagnosis with COPD in primary care record. | |
| neurological | Flag indicating diagnosis with Neurological in primary care record. | |
| dementia | Flag indicating diagnosis with Dementia in primary care record. | |
| autism | Flag indicating diagnosis with Autism in primary care record. | |
| ADHD | Flag indicating diagnosis with ADHD in primary care record. | |
| gp_antidepressant_rx_12 | number of antidepressants prescribed in the last 12 months – defined as BNF codes starting ‘0403’ | |
| **Activity variables** | |  |
| **Secondary care data** | |  |
| **Variable** | **Description** |  |
| admissions_electives_12 | number of elective admissions in the last 12 months |  |
| Admissions_electives_cost_12 | total cost of elective admissions in the last 12 months based on HRG tariff |  |
| admissions_emergency_12 | number of emergency admissions in the last 12 months |  |
| Admissions_electives_cost_12 | total cost of emergency admissions in the last 12 months based on HRG tariff |  |
| aae1a2_attend_12 | number of Accident and Emergency (A&E) attendances in type 1 and 2 facilities the last 12 months |  |
| aae_attendances_cost_12 | total cost of A&E attendances in type 1 and 2 facilities in the last 12 months based on HRG tariff |  |
| aae_selfharm_12 | A&E attendances for in the last 12 for self-harm |  |
| aae_alcohol_12 | A&E attendances for in the last 12 for alcohol misuse |  |
| aae_eating_12 | A&E attendances for in the last 12 for eating disorders |  |
| aae_substance_12 | A&E attendances for in the last 12 for substance abuse |  |
| aae_mental_health_12 | Count of A&E attendances for mental health reasons in the last 12 months – mental health reasons are: self-harm, alcohol and/substance abuse, eating disorder and any other mental health conditions. |  |
| emadm_selfharm_12 | Emergency admissions for in the last 12 for self-harm |  |
| emadm_eating_12 | Emergency admissions for in the last 12 for Eating disorders |  |
| emadm_substance_12 | Emergency admissions for in the last 12 for substance abuse |  |
| emadm_alcohol_12 | Emergency admissions for in the last 12 for alcohol abuse |  |
| emadm_otherpsych_12 | Emergency admissions for in the last 12 for other psychiatric reasons. |  |
| **Mental health dataset (MHDS** [1] **or similar)** | |  |
| **variable** | **description** |  |
| mh_referrals_tot_12 | Total number of referrals to mental health services in the last 12 month |  |
| mh_contacts_tot_12 | Total number of referrals to mental health services in the last 12 month |  |
| ref_service_autism_12 | number referrals to mental health services in the last 12 months service for autism |  |
| ref_service_learning_neurodevelop_12 | number referrals to mental health services in the last 12 months service learning/neurodevelopmental |  |
| ref_service_learning_forensic_12 | number referrals to mental health services in the last 12 months service forensic |  |
| ref_service_neurodevelopment_12 | number referrals to mental health services in the last 12 months service neurodevelopmental |  |
| ref_service_sp_access_12 | number referrals to mental health services in the last 12 months single point of contact |  |
| ref_service_severe_12 | number referrals to mental health services in the last 12 months severe mental health problems |  |
| ref_service_psychosis_early_12 | number referrals to mental health services in the last 12 months for psychosis |  |
| cont_service_autism_12 | number contacts with mental health services in the last 12 months service autism |  |
| cont_service_community_12 | number contacts with mental health services in the last 12 months service in the community |  |
| cont_service_eating_12 | number contacts with mental health services in the last 12 months service eating |  |
| cont_service_learning_neurodevelop_12 | number contacts with mental health services in the last 12 months service neurodevelopmental |  |
| cont_service_learning_forensic_12 | number contacts with mental health services in the last 12 months service forensic |  |
| cont_service_neurodevelopment_12 | number contacts with mental health services in the last 12 months service neurodevelopmental |  |
| cont_other_12 | number contacts with mental health services in the last 12 months service other |  |
| cont_service_perinatalparenting_12 | number contacts with mental health services in the last 12 months service perinatal\parenting |  |
| cont_service_personality_12 | number contacts with mental health services in the last 12 months service personality disorder |  |
| cont_service_psychosis_early_12 | number contacts with mental health services in the last 12 months service early psychosis |  |
| cont_service_roughsleeping_12 | number contacts with mental health services in the last 12 months service rough sleeping |  |
| cont_service_severe_12 | number contacts with mental health services in the last 12 months service severe mental health |  |
| cont_service_substance_12 | number contacts with mental health services in the last 12 months service substance abuse |  |
| **Community services (CSDS**[2] **or similar) and adult social care dataset**[3]**.** | |  |
| **variable** | **description** |  |
| cs_contacts_tot_12 | total number of contacts to community health services in the last 12 months (including health visitor or midwifery services) |  |
| cs_cont_service_healthvisitormidwife_12 | total number of contacts to health visitor or midwifery services in the last 12 months |  |
| asc_services_12 | total number of adult social care service contacts in the last 12 months |  |
| asc_requests_12 | total number of requests for social care support in the last 12 months |  |
| asc_service_reason_learning_12 | number of contacts with adult social care services in the last 12 months for reason learning disability |  |
| asc_request_reason_learning_12 | number of referrals to adult social care services in the last 12 months for reason learning disability |  |
| asc_request_reason_carer_12 | number of referrals to adult social care services in the last 12 months for reason carer support |  |

1. **Individual derived variables**

Based on the variables in table 1 a number of derived variables are calculated as given in table 2. In the description of the derivations we use some pseudocode and we follow this notation:

- Names of derived variables are highlighted in grey
- Pseudocode for definitions is highlighted in grey
- = means assignment or definition
- == means equality
- | is the same as the logic operator OR
- & is the same as the logic operator AND
- as.numeric() is used in this code to change the logic binary flag into a number (1/0), where 1 is the same as TRUE and 0 is the same as FALSE.

Table 2. Individual derived variables

| **variable** | **description** | **Pseudo-code** |
| --- | --- | --- |
| child | Flag indicating a child | As.numeric(age<18) |
| Adult | Flag indicating a adult | As.numeric(age>17) |
| dep | Flag indicating resident is living in the most income deprived areas (inc_child_score > 80^th^ percentile | as.numeric(inc_child_score >p80(inc_child_score)) |
| num_ltc | Number of long term conditions | The sum of diagnostic flags given in table1:  num_ltc= cancer + asthma + ckd + cld + copd + cvd + cmhp + rheumatological+  + dementia + diabetes + epilepsy + neurological  + smi |
| ltc | Flag for at least 1 LTC. | As.numeric(ltc>0) |
| num_phys_ltc | Number of physical health conditions | num_phys_ltc = cancer + asthma + ckd + cld + copd + cvd + rheumatological+  diabetes + epilepsy + neurological+dementia |
| num_mental_ltc | Number of mental health conditions | cmhp + smi |
| ltc_adult | Flag for long term condition in adult | ltc_adult =as.numeric(num_ltc>0 & age>17) |
| phys_ltc_adult | Flag for physical long term condition in adult | ltc_phys_adult =as.numeric(num_phys_ltc>0 & age>17) |
| mental_ltc_adult | Flag for mental long term condition in adult | ltc_mental_adult =as.numeric(num_mental_ltc>0 & age>17) |
| ltc_child | Flag for long term condition in child | ltc_child =as.numeric(num_ltc>0 & age<18) |
| phys_ltc_child | Flag for physical long term condition in child | ltc_phys_child =as.numeric(num_phys_ltc>0 & age<18) |
| mental_ltc_child | Flag for mental long term condition in child | ltc_mental_child =as.numeric(num_mental_ltc>0 & age<18) |
|  |  |  |
| high_ae | Flag indicating greater than 5 A&E attendances in the past 12 months | as.numeric (ae1a2_attend_12>5) |
| Learning_disability | Flag indicating if the person has a learning disability/ difficulty or neurodevelopmental condition, derived from primary care and mental health datasets. Defined as anyone who is either flagged with learning disability of neurodevelopmental condition in primary care or has had contact or been referred to mental health and learning disability services or social services for learning disability/difficulty or neurodevelopmental condition | Learning_disability= as.numeric( cont_service_autism_12 + cont_service_neurodevelopment_12 + cont_service_learning_forensic_12 + cont_service_learning_neurodevelop_12+ ref_service_learning_neurodevelop_12+  ref_service_autism_12 + ref_service_neurodevelopment_12 + ref_service_learning_forensic_12 +  ref_reason_autism_12+  ref_reason_neurodevelopmental_12>0 \| learning_disability_pc==1\|Autism==1  ADHD==1\|asc_service_reason_learning_12>0\|asc_request_reason_learning_12>0) |
| asc_user | Defined as anyone with either a contact or a referral to adult social care | asc_user=as.numeric(asc_services_12>0 \| asc_requests_12>0 ) |
| Substance_abuse | A flag indicating if a person has any record of substance abuse in primary care, mental health services or secondary care | substance_abuse= as.numeric((  substance_misuse_gpl==1\|  ref_reason_substance_12>0\|ref_reason_substance_12>0 \|  cont_service_substance_12>0\| aae_substance_12>0\|  aae_alcohol_12>0\| emadm_alcohol_12>0\|emadm_substance_12>0) |
| mental_mhds_contacts | Mental health MHDS contacts, excluding those due to learning difficulties / disabilities / neurodevelopmental conditions | mental_mhds_contacts=mh_contacts_tot_12 -cont_service_autism_12 -cont_service_learning_forensic_12 -cont_service_learning_neurodevelop_12 -  cont_service_neurodevelopment_12 |
| cs_exc_mwhv | Community service contacts excluding midwifery and health visiting | cs_exc_mwhv= cs_contacts_tot_12-cs_cont_service_healthvisitormidwife_12 |
| Any_mh | A flag indicating any mental health problem from across all datasets | any_mh=as.numeric(cmhp+smi+mental_mhds_contacts+ref_reason_depression_12+ref_reason_anxiety_12+ref_reason_psychosis_12+ref_service_psychosis_early_12+ref_service_severe_12+cont_service_psychosis_early_12+ cont_service_severe_12+gp_antidepressant_rx_12+substance_abuse+emadm_selfharm_12+emadm_eating_12+emadm_substance_12+emadm_alcohol_12 +  emadm_otherpsych_12+aae_selfharm_12+aae_alcohol_12+aae_eating_12+aae_substance_12>0) |
| Any_mh_adult | A flag indicating any mental health problem in adults | As.numeric(any_mh==1 & age>17) |
| child_prob | A flag indicating if a child has a long term condition, learning disability, mental health problem, social care problem, or is accessing community services other than midwifery or health visiting. | child_prob=as.numeric(ltc+any_mh+learning_disability+ cs_exc_mwhv + child_looked_after>0)] |

1. **Individual exclusion criteria**

Individuals in table 1 were then excluded if they:

1. Missing data on UPRN, age or sex. (UPRN==NA | age==NA|sex==NA)
2. Were living in a nursing home (NursingCareHomeFlag==1)
3. Were dead (deceased==1, or a date of death was otherwise recorded)
4. **Aggregation at household level**

Each of the numerical variables in table 1 and derived variables in table 2 are then summed for each household. Where a household is defined as people with the same UPRN, i.e living at the same address. Below we apply the prefix nump_ before variables in tables 1 & 2 to indicate the household sum for each variable. For example nump_child is the number of children in the household, nump_ltc_adult is the number of adults with a long term condition etc.

1. **Cost estimation**

The costs of health and social care activity for each household was then approximately estimated applying the following unit costs. For some activity and characteristics we did not have access to sufficiently granular data on activity and costs to directly estimates of the costed specific activity. In these cases we have applied an average cost derived from external data sources, recognising that the activity for a specific individual may differ to a large extent from these average costs. These costs were however only used to identify a segment of households that have relatively high intensity use (the upper quartile) and are therefore reasonable approximations for this use.

Table 3: Cost estimates applied to different service types.

| **Variable (A)** | **Activity** | **Annual cost weights applied (B)** | **Unit** | **Notes** | **Source** |
| --- | --- | --- | --- | --- | --- |
| **Primary Care** | | | | | |
| Nump_cancer | Cancer | £478 | Annual per person | These are only primary care costs (costed for main long term conditions). We compared these costs to costing of activity for selected diseases in primary care using CPRD data (see appendix 1) | Kasteridis et al. (pg.36-51)[4] |
| Nump_asthma | Asthma | £312 |  |  |  |
| Nump_ckd | CKD | £745 |  |  |  |
| Nump_copd | COPD | £687 |  |  |  |
| Nump_cvd | CVD | £644 |  |  |  |
| Nump_cmhp | Common Mental Health Problems | £372 |  |  |  |
| Nump_Rheumatological | Rheumatological | £593 |  |  |  |
| Nump_dementia | Dementia | £757 |  |  |  |
| Nump_diabetes | Diabetes | £604 |  |  |  |
| Nump_epilepsy | Epilepsy | £358 |  |  |  |
| Nump_Neurological | Neurological | £644 |  |  |  |
| Nump_smi | Severe Mental illness | £315 |  |  |  |
| **Children’s social care** | | | | | |
| Nump_child_looked_after | Child looked after | £49,169 | Annual per child looked after | Others measures of children’s social care were not available.  Calculated at the total expenditure on children looked after in Liverpool/ number of children looked after = 73263000/1490 | Data reported for Liverpool CC on LG Inform[5] |
| **Adult Social Care** | | | | | |
| Nump_Asc_user | Adult Social Care activity | £ 16,535 | Annual per person | Statutory budget return £210 million total expenditure on adult social care with 12,700 short and long term clients in 2021. | NHS Digital[6] |
| Nump_Learning_disability | Learning disability | £ 3294 | Annual per person | Assuming 1 day-care session per week. (in addition to social care costs) | PSSRU: Unit Costs of Health and Social Care 2021[7] |
| **Secondary care** | | | | | |
| Nump_EmergencyAdmissions_cost_12 | Emergency Admissions | Varied by activity based on Tariff costs | Per unit of activity |  | National Tariff[8] |
| Nump_ElectiveAdmissions_cost_12 | Elective admissions |  |  |  |  |
| Nump_AAEAttendances_cost_12 | Accident and Emergency |  |  |  |  |
| **Mental health service** | | | | | |
| Nump_mh_contacts_tot_12 | Mental health service contact | £342 | Per contact | Average national reference costs for mental health services in 2021 | National reference costs[9] |
| Nump_cs_contacts_tot_12 | Community services | £80 | Per contact | Average national reference costs for community services in 2021 | National reference costs[9] |

The total health and social care cost was then calculated as the number of people with each characteristic (where unit was per person) or number of contacts (where the unit is per contact) (column A), multiplied by the costs (column B), apart from for secondary care data where costs were available in the original data source and these were used directly. Costs for each area of activity were the summed to give then total household costs (total_cost).

1. **Household exclusion criteria**

Households were excluded if they do not contain any children (nump_child==0) or if they have more than 10 members, i.e. there are 10 or more people living at the same address, as this is likely to reflect institutions (e.g. care homes) or houses of multiple occupancy, rather than individual households.

1. **Household derived variables**

Table 4: Definition of household level derived variables.

| **variable** | **description** | **Pseudo-code definition** |
| --- | --- | --- |
| Lone_parent | Flag indicating a potential lone parent, defined as a household with children and only 1 adult | as.numeric(nump_adult==1 & num_child>0) |
| Num_serv | Number of different services used by the household, where services are:  Admitted patient care, Accident and emergency, primary care(having a long term condition), mental health services, community health services, adult social care, children’s social care (child looked after). | num_serv=as.numeric((Nump_EmergencyAdmissions_cost_12  + Nump_ElectiveAdmissions_cost_12  )>0)+as.numeric(Nump_AAEAttendances_cost_12>0)+  as.numeric(nump_ltc)>0) +  as.numeric(Nump_mh_contacts_tot_12>0)+  as.numeric(Nump_cs_contacts_tot_12>0)+  as.numeric(nump_asc_user>0)+  as.numeric(Nump_child_looked_after >0)) |
| social | Flag indicating households with at least one indicator of potential social problems | As.numeric(Substance_abuse+nump_homeless+lone_parent+dep+ Nump_child_looked_after+as.numeric(num_serv>2)>0) |
| High_cost | Flag identifying households with high overall cost weighted utilisation – in the upper quartile | As.numeric(total_cost>=p75(total_cost) |

1. **Definition of complex households**

Complex households are then defined as households where there is are adult(s) with both mental and physical health conditions, a child with a health or social care problem and the household has at least 1 indicator of social problems and is in the upper quartile for overall high cost-weighted utilisation.

Table 5: Programmatic definition of households with complex needs.

| **variable** | **description** | **Pseudo-code definition** |
| --- | --- | --- |
| complex_hh | Flag identifying households with complex needs. | complex_hh:=as.numeric(nump_phys_ltc_adult >0 & nump_Any_mh_adult >0 & nump_child_prob>0 & Social==1 & high_cost==1) |

References

1 Mental Health Services Data Set (MHSDS). NHS Digit. https://digital.nhs.uk/data-and-information/data-collections-and-data-sets/data-sets/mental-health-services-data-set (accessed 15 March 2024)

2 Community Services Data Set (CSDS). NHS Digit. https://digital.nhs.uk/data-and-information/data-collections-and-data-sets/data-sets/community-services-data-set (accessed 15 March 2024)

3 Adult Social Care Client Level Data - NHS Digital. https://digital.nhs.uk/data-and-information/data-collections-and-data-sets/data-sets/adult-social-care-client-level-data (accessed 15 March 2024)

4 Kasteridis P, Street A, Dolman M, *et al.* The importance of multimorbidity in explaining utilisation and costs across health and social care settings: evidence from South Somersets Symphony Project. *Work Pap*. Published Online First: February 2014.

5 Association LG. Total Expenditure - Children Looked After in Liverpool. https://lginform.local.gov.uk/reports/lgastandard?mod-metric=%3C7592M%3E&mod-area=E08000012&mod-group=AllMetropolitanBoroughLaInCountry_England&mod-type=namedComparisonGroup&mod-period=4 (accessed 19 November 2023)

6 Adult Social Care Statistics in England: An overview by region and local authority. NHS Digit. https://digital.nhs.uk/data-and-information/data-tools-and-services/data-services/adult-social-care-data-hub/dashboards/adult-social-care-statistics-in-england-an-overview (accessed 19 November 2023)

7 Unit Costs of Health and Social Care 2021 | PSSRU. https://www.pssru.ac.uk/project-pages/unit-costs/unit-costs-of-health-and-social-care-2021/ (accessed 19 November 2023)

8 NHS England » National Tariff. https://www.england.nhs.uk/pay-syst/national-tariff/ (accessed 19 November 2023)

9 NHS England » 2021/22 National Cost Collection Data Publication. https://www.england.nhs.uk/publication/2021-22-national-cost-collection-data-publication/ (accessed 19 November 2023)
