# Supplementary material for: Identifying households with children who have complex needs: a segmentation model for integrated care systems
Source: BMC Health Serv Res. 2025 Jan 27;25:152. doi: 10.1186/s12913-024-12100-x (PMC11773761; doi:10.1186/s12913-024-12100-x)
Supplement: Supplementary file 5 — Supplementary Material 5. [file 12913_2024_12100_MOESM5_ESM.docx]

**Identifying households with children who have complex needs: a segmentation model for integrated care systems**

**Supplemental materials: Co-use of different types of services**

In Appendix B we have described the different types of services provided in several sectors and separate organisations. To summarise, we combined data of service use in:

1. Primary care,(1)
2. Secondary elective care, (2)
3. Secondary emergency,(2,3)
4. Mental health care (emergency and routine),(4)
5. Community health care ,(5),
6. Adult social care all,(6)

The aim of this project was to support integration of services. To provide integrated services proportional to actual need, or better demand we aimed to understand the pattern of service use of households who experience complex needs and that therefore have contacts with multiple services. We know from the literature and from the voices of lived experts, that households find it challenges to access multiple separate services for several needs and/or households members. To guide integration with evidence, we looked at co-use of services to discover which services would be more useful to integrate.

Figure 1 shows the co-use of several service types.

Figure 1: Pattern of co-use of service (as percentage of households using a specific combination of two different services), comparing all other households with children (L) and households with complex needs (R) in the English region of Cheshire and Merseyside.


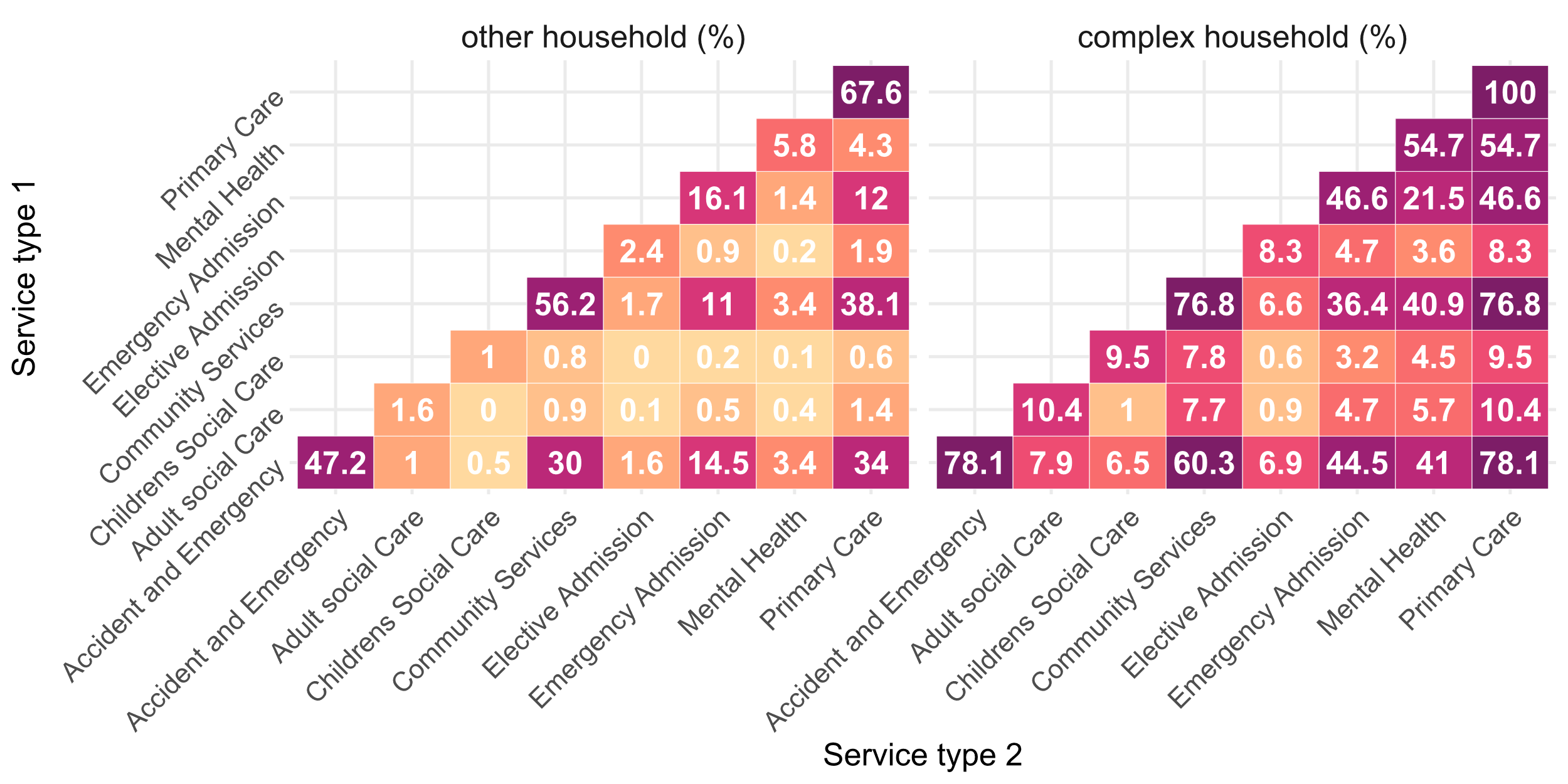


The two matrices show combinations of use of two types of services. The services we considered are listed along the axes, there are 8 groups: 1-Accident and Emergency ( also A&E, that is Emergency Department or Emergency Room in the UK), 2-Adult Social Care,3-Children Social Care, 4-Community Services, 5-Elective Admissions, 6-Emergency Admissions, 7-Mental Health and 8-Primary Care. The diagonal in the matrix indicates the percentage of households who use any of the 8 service types take in isolation, while the off-diagonal values show the percentage of households who use two services in conjunction, for example, the right hand column shows on the top value that 100% of complex households use primary care, the value below shows that 55% use primary care and mental health care services, 46% use primary care and had emergency admissions to hospital, 77% used community services in conjunction with primary care and 78% visited an emergency department in conjunction with using primary care.

The matrix on the left shown the level of co-use of services by all other households with children in the region of the UK where this study took place. The matrix on the right shown the level of co-use of services in households with complex needs.

References

1. NHS England » Purpose of the GP electronic health record [Internet]. [cited 2024 Oct 28]. Available from: https://www.england.nhs.uk/long-read/purpose-of-the-gp-electronic-health-record/

2. NHS England Digital [Internet]. [cited 2024 Oct 28]. Secondary Uses Service (SUS). Available from: https://digital.nhs.uk/services/secondary-uses-service-sus

3. NHS England Digital [Internet]. [cited 2024 Oct 28]. Emergency Care Data Set (ECDS). Available from: https://digital.nhs.uk/data-and-information/data-collections-and-data-sets/data-sets/emergency-care-data-set-ecds

4. NHS England Digital [Internet]. [cited 2024 Oct 28]. Mental Health Services Data Set (MHSDS). Available from: https://digital.nhs.uk/data-and-information/data-collections-and-data-sets/data-sets/mental-health-services-data-set

5. NHS England Digital [Internet]. [cited 2024 Oct 28]. Community Services Data Set (CSDS). Available from: https://digital.nhs.uk/data-and-information/data-collections-and-data-sets/data-sets/community-services-data-set

6. NHS Arden&GEM. Adult Social Care Client Level Data. [cited 2024 Jun 7]. Adult Social Care Client Level Data. Available from: https://www.ardengemcsu.nhs.uk/adult-social-care-client-level-data/

7. Office for National Statistics. ons.gov.uk. [cited 2024 Jun 7]. Mortality statistics in England and Wales QMI - Office for National Statistics. Available from: https://www.ons.gov.uk/peoplepopulationandcommunity/birthsdeathsandmarriages/deaths/methodologies/mortalitystatisticsinenglandandwalesqmi

8. Department for Levelling Up, Housing and Communities. GOV.UK. [cited 2024 Jun 7]. English indices of deprivation 2019. Available from: https://www.gov.uk/government/statistics/english-indices-of-deprivation-2019
